# Marine Antimicrobial Peptide Epinecidin-1 Inhibits Proliferation Induced by Lipoteichoic acid and Causes cell Death in non-small cell lung cancer Cells via Mitochondria Damage

**DOI:** 10.1007/s12602-023-10130-1

**Published:** 2023-07-31

**Authors:** Hsin-Hsien Yu, Luo-Yun Wu, Pei-Ling Hsu, Chu-Wan Lee, Bor-Chyuan Su

**Affiliations:** 1 Division of General Surgery, Department of Surgery, Wan Fang Hospital, Taipei Medical University, Taipei, Taiwan; 2 Division of General Surgery, Department of Surgery, School of Medicine, College of Medicine, Taipei Medical University, Taipei, Taiwan; 3 School of Medicine, College of Medicine, Taipei Medical University, Taipei, Taiwan; 4 Department of Anatomy, School of Medicine, College of Medicine, Kaohsiung Medical University, Kaohsiung, 80708 Taiwan; 5 Department of Medical Research, Kaohsiung Medical University Hospital, Kaohsiung, 80708 Taiwan; 6 Department of Nursing, National Tainan Junior College of Nursing, 78, Section 2, Minzu Road, West Central District, Tainan, 70007 Taiwan; 7 Department of Anatomy and Cell Biology, School of Medicine, College of Medicine, Taipei Medical University, Taipei, Taiwan; 8 Graduate Institute of Medical Sciences, College of Medicine, Taipei Medical University, Taipei, Taiwan

**Keywords:** Marine antimicrobial peptide, Epinecidin-1, Non-small-cell lung cancer, Proliferation, Lipoteichoic acid

## Abstract

Non-small cell lung cancer (NSCLC) is among the deadliest cancers worldwide. Despite the recent introduction of several new therapeutic approaches for the disease, improvements in overall survival and progression-free survival have been minimal. Conventional treatments for NSCLC include surgery, chemotherapy and radiotherapy. Except for surgery, these treatments can impair a patient’s immune system, leaving them susceptible to bacterial infections. As such, *Staphylococcus aureus* infections are commonly seen in NSCLC patients receiving chemotherapy, and a major constituent of the *S. aureus* cell surface, lipoteichoic acid (LTA), is thought to stimulate NSCLC cancer cell proliferation. Thus, inhibition of LTA-mediated cell proliferation might be a useful strategy for treating NSCLC. Epinecidin-1 (EPI), a marine antimicrobial peptide, exhibits broad-spectrum antibacterial activity, and it also displays anti-cancer activity in glioblastoma and synovial sarcoma cells. Furthermore, EPI has been shown to inhibit LTA-induced inflammatory responses in murine macrophages. Nevertheless, the anti-cancer and anti-LTA activities of EPI and the underlying mechanisms of these effects have not been fully tested in the context of NSCLC. In the present study, we demonstrate that EPI suppresses LTA-enhanced proliferation of NSCLC cells by neutralizing LTA and blocking its effects on toll-like receptor 2 and interleukin-8. Moreover, we show that EPI induces necrotic cell death via mitochondrial damage, elevated reactive oxygen species levels, and disrupted redox balance. Collectively, our results reveal dual anti-cancer activities of EPI in NSCLC, as the peptide not only directly kills cancer cells but it also blocks LTA-mediated enhancement of cell proliferation.

## Introduction

While chemotherapy can initially curb cancer progression in many patients, the frequent development of chemoresistance hinders its sustained efficacy [1] in a wide variety of cancers, including breast, colorectal, lung and other cancer types [2]. In addition, chemotherapy can greatly weaken a patient’s immune system by reducing the number of leukocytes, rendering the patient more susceptible to bacterial infections [3]. *Staphylococcus aureus* is the most commonly identified gram-positive bacterial cause of pneumonia in lung cancer patients [4, 5], and 76.3% of *S. aureus* strains isolated from cancer patients are resistant to methicillin (MRSA: methicillin-resistant *S*. *aureus*) [6]. Since MRSA infection is correlated with non-small cell lung cancer (NSCLC) cell metastasis [7], vancomycin is routinely prescribed for NSCLC patients with MRSA [8]. Yet, NSCLC patients who have received vancomycin have shorter progression-free survival and overall survival than those who have not [9], potentially due to vancomycin-associated renal toxicity [10]. Furthermore, antibiotic-induced release of pathogen-associated molecular patterns (PAMPs) from bacteria may contribute to cancer progression [11]. Previous work has shown that released PAMPs can stimulate a variety of oncogenic processes, including proliferation, migration, invasion and angiogenesis [12, 13]. For instance, lipopolysaccharide from *Escherichia coli* was shown to promote NSCLC cell proliferation, migration and invasion [12, 13], while another recent study revealed that lipoteichoic acid (LTA) from *S. aureus* can promote the development of lung cancer [14]. In particular, it was shown that LTA induces proliferation of NSCLC cell lines by stimulating secretion of interleukin-8 (IL-8) [14]. Thus, agents that can neutralize LTA might help to alleviate lung cancer progression, especially in patients who are prone to gram-positive bacterial infections due to chemotherapy-induced immunosuppression.

Epinecidin-1 (EPI) is a synthesized antimicrobial peptide that was originally identified from orange-spotted grouper (*Epinephelus coioides*) [15]. The peptide is known to exhibit broad-spectrum antibacterial activity against *Pseudomonas aeruginosa* [16], *Vibrio vulnificus* [17], *Helicobactor pylori* [18], *E. coli* [18], and MRSA [19]. Moreover, we recently found that EPI is able to modulate LTA-induced inflammation in murine Raw 264.87 macrophages by preventing toll-like receptor (TLR)2 internalization [20]. In addition to its antibacterial and immunomodulatory activities, EPI also exhibits anti-cancer activity in synovial sarcoma [21] and glioblastoma cells as an inducer of oxidative stress [22]. A previous report demonstrated that EPI has anti-lung cancer activity [23]. However, the underlying cytotoxic mechanisms are not fully addressed. In this study, we tested whether the known multifaceted activities of EPI might contribute to its anti-lung cancer function.

## Materials and Methods

### Reagents and Peptides

EPI (H-GFIFHIIKGLFHAGKMIHGLV-OH) and scrambled EPI (SE; H- VGHHIIKLGGAIGFFLFMIKH-OH) were synthesized by GL Biochem (China) [22]. Trypan Blue solution, MitoTracker Red CMXRos (MitoTracker), and tetramethylrhodamine ethyl ester (TMRE) were purchased from ThermoFisher (USA). MTS was purchased from Promega (USA). LTA from *S. aureus*, DCFDA, DHE, trolox TRO, and TEMPO were purchased from Merck (Germany). Propidium iodide (PI) was purchased from ThermoFisher (USA). Z-VAD-FMK was purchased from Cell Signaling Technology (USA).

### Cell Culture

The A549 NSCLC cell line was purchased from American Type Culture Collection (USA) and maintained in Dulbecco’s Modified Eagle Medium (DMEM; ThermoFisher Scientific, USA) supplemented with 10% fetal bovine serum (Peak Serum, Inc, USA) and penicillin-streptomycin (Sartorius, Germany).

### Cell Proliferation and Viability Assays

Cell proliferation was monitored with the MTS and trypan blue exclusion assays. In addition, two cell proliferation markers were analyzed, including cyclin D1 [24] and phospho-Histone H3 [25]. MTS and trypan blue exclusion assays were performed as described previously [21]. The trypan blue exclusion assay was used to calculate cell numbers and cell viability. In order to calculate relative cell proliferation ratios, the viable cell numbers from LTA/EPI groups were divided by the viable cell numbers from control groups. Cell viability was calculated as the viable cell number divided by the total cell numbers. Cell proliferation markers (cyclin D1 and phospho-Histone H3) were detected by western blot. The PI uptake assay was performed to detect necrotic cell death. Briefly, cells were treated as indicated and then loaded with PI (1 µg/ml). After rinsing with PBS three times, the cells were imaged by fluorescence microscopy (Motic, Spain).

### Western blot Analysis and Antibodies

Cells were lysed with RIPA buffer (Merck, Germany), and proteins were separated on a gradient gel before transfer onto a PVDF membrane (Cytiva, USA). Thereafter, the membranes were probed with indicated antibodies. Cyclophilin A, cyclin D1, phospho-Histone H3, toll-like receptor 2 (TLR2), interleukin (IL)-8, catalase, UCP2 and β-actin antibodies were purchased from Cell Signaling Technology (USA).

### Mitochondrial Function Assay and ROS Analysis

In order to examine mitochondrial function, cells were treated as described and then loaded with MitoTracker (100 nM) and TMRE (100 nM) for 20 min. Thereafter, cells were rinsed with PBS three times. The fluorescence intensities of TMRE and MitoTracker were observed and recorded under fluorescence microscopy (Motic, Spain). Intracellular ROS was evaluated with fluorescent ROS probes, DCFDA (10 µM) and DHE (20 µM). Cells were stained with DCFDA or DHE for 20 min and then rinsed with PBS three times. The fluorescence intensities of DCFDA and DHE were observed and recorded under fluorescence microscopy (Motic, Spain).

### Surface TLR2 Analysis

In order to detect TLR2 on the cell surface, cells were first treated with EPI and LTA as indicated. Then, cells were incubated with FITC-TLR2 antibody (ThermoFisher Scientific, USA) for 1 h at 4 °C. Next, cells were washed with PBS, and the levels of cell surface TLR2 were assessed by flow cytometry (Beckman Coulter, USA).

### LTA/Epi Binding Assay

The LTA/EPI binding assay was performed as described in a previous publication with minor modifications [26]. Briefly, LTA-coated 96-well plates were blocked with 1% bovine serum albumin (ThermoFisher Scientific, USA) for 1 h at room temperature (RT). Next, EPI (0–10 µg/ml) was added to each well, and the plates were incubated for another 1 h at 37 °C. The wells were washed with PBS. Thereafter, wells were incubated with EPI antibody (1:100) and secondary antibody (HRP rabbit anti-mouse IgG; 1:1000) for 1 h at RT. LTA/Epi binding activity was detected using TMB substrate.

### Statistical Analysis

All experiments were performed at least three times. Statistical significance was assessed by Student’s *t*-test or one-way ANOVA. *P* values smaller than 0.05 were considered statistically significant.

## Results

### LTA Stimulates Proliferation of A549 Cells

**Fig. 1:**
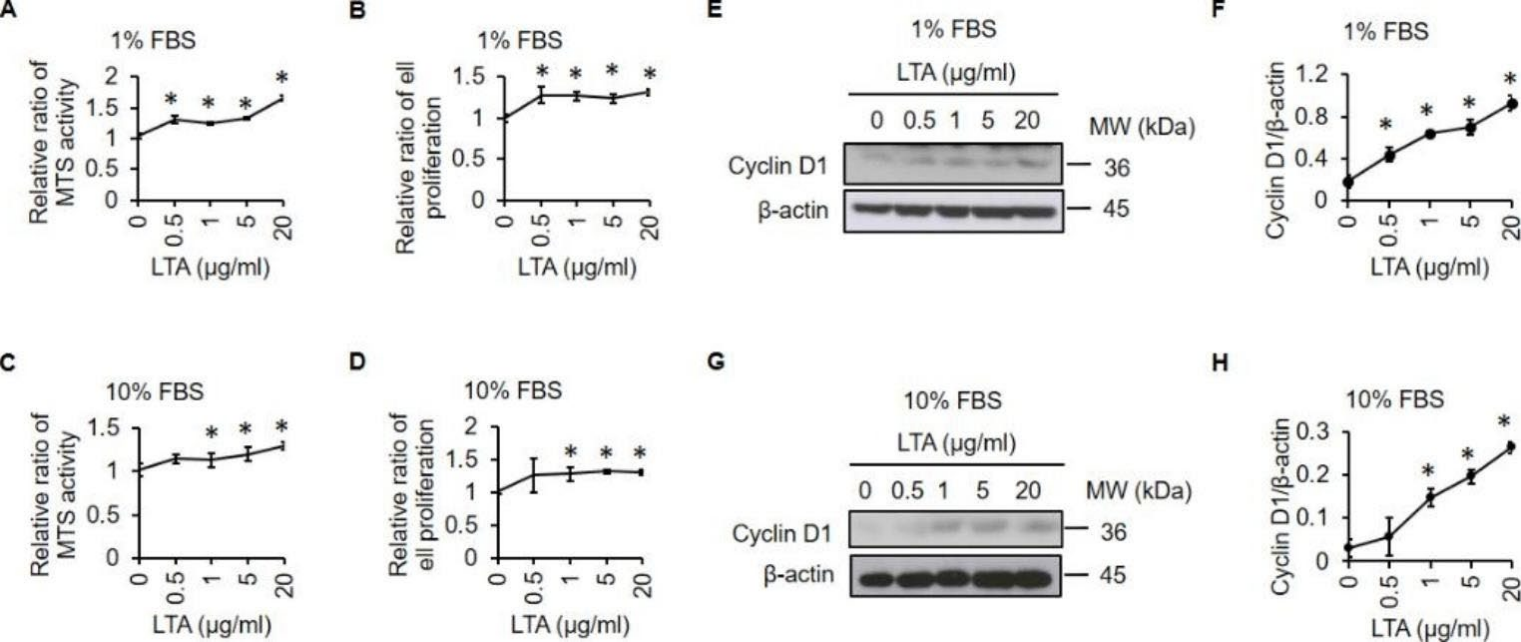
**LTA induces proliferation in A549 cells.** Cells were treated with increasing doses of LTA (0, 0.5, 1, 5 and 20 µg/ml) in 1% FBS medium **(A, B, E and F)** or 10% FBS medium **(C, D, G and H)** for 24 h. Cell viability was determined by the MTS assay **(A, C)** and trypan blue exclusion assay **(B, D)**. Western blot analyses showing the protein expression of cyclin D1 **(E, G)**. The band intensity of cyclin D1 was measured by ImageJ **(F, H)**. *: *P* < 0.05. All experiments were independently performed at least three times

A previous report showed that LTA can stimulate proliferation of human NSCLC cells [14]. Since the concentration of serum in culture medium is a key determinant of LTA-induced inflammatory strength [27], we were curious whether serum levels might also affect LTA-mediated enhancement of NSCLC cell proliferation. Therefore, we assessed the proliferative effects of LTA on human NSCLC A549 cells in low serum (1%) and normal serum (10%) conditions. Cell proliferation was assessed by the MTS assay (Fig. 1A and C) and trypan blue exclusion assay (Fig. 1B and D). Similar to the findings from a previous study [14], MTS signal and cell numbers were dose-dependently increased by LTA in both low serum and normal serum conditions. In addition, LTA treatments increased the levels of cell proliferation marker cyclin D1 (Fig. 1E-H) in both low serum and normal serum conditions. These results indicate that LTA stimulates NSCLC cell proliferation in both low and normal serum conditions.

### EPI Attenuates LTA-induced cell Proliferation

**Fig. 2:**
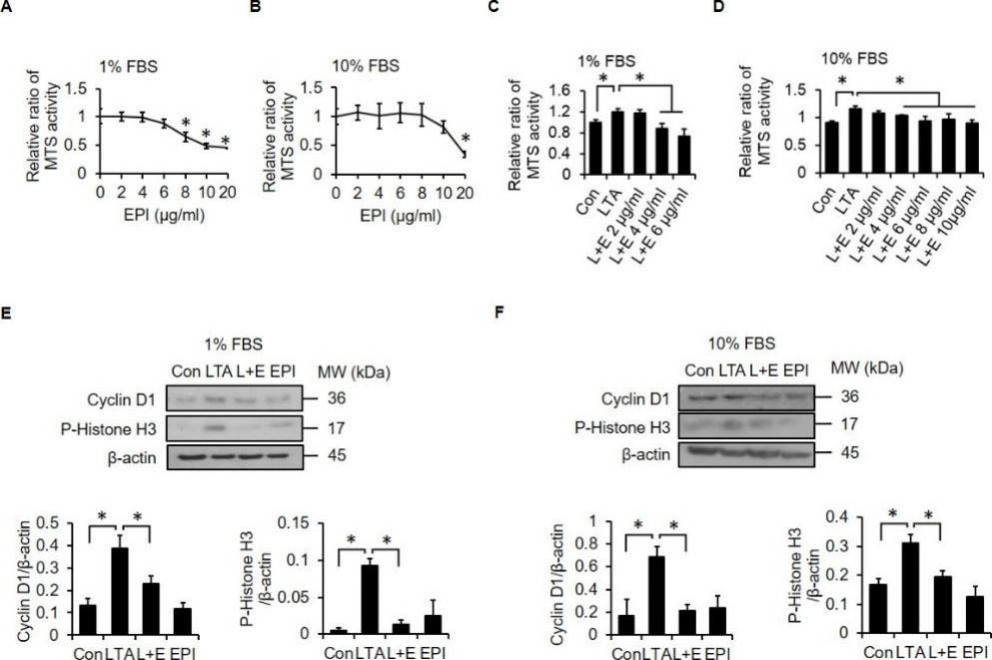
**EPI inhibits LTA-induced proliferation of A549 cells.** Cells were treated with increasing doses of EPI (0, 2, 4, 6, 8, 10 and 20 µg/ml) in 1% FBS **(A)** or 10% FBS **(B)** medium for 24 h. Cell viability was measured using MTS activity assay. Cells were treated with LTA (20 µg/ml) alone or with the indicated doses of EPI in combination with LTA in 1% FBS **(C)** or 10% FBS **(D)** media for 24 h. Cell viability was measured with the MTS assay. Cells were treated with EPI (6 µg/ml) alone, LTA (20 µg/ml) alone or EPI in combination with LTA in 1% FBS **(E)** or 10% FBS **(F)** media for 24 h. Western blot analyses and relative densitometric graphs show the protein expression levels of cyclin D1 and phospho-Histone H3. *: *P* < 0.05 compared to the control or between the indicated groups. Con: control; L + E: LTA + EPI. All experiments were independently performed at least three times

Since the activities of antimicrobial peptides are often influenced by the presence of serum components [28, 29], we assessed the effects of EPI on cell proliferation in both low and normal serum conditions. In the low serum condition, signal from the MTS assay was significantly reduced when cultures were treated with at least 8 µg/ml EPI (Fig. 2A). In the normal serum condition, MTS signal was reduced when cells were exposed to at least 20 µg/ml EPI (Fig. 2B). Next, we determined whether EPI suppresses LTA-mediated enhancement of proliferation in low and normal serum conditions. We observed that EPI attenuated LTA-induced cell proliferation in both low (Fig. 2C) and normal serum conditions (Fig. 2D), with a lowest effective dose of 4 µg/ml EPI. In addition, LTA-induced upregulation of proliferation markers (cyclin D1 and phospho-Histone H3) was suppressed by EPI in both low (Fig. 2E) and normal serum (Fig. 2F) conditions.

### EPI Blocks LTA-induced cell Proliferation by Suppressing TLR2 Internalization and IL-8 Upregulation

**Fig. 3:**
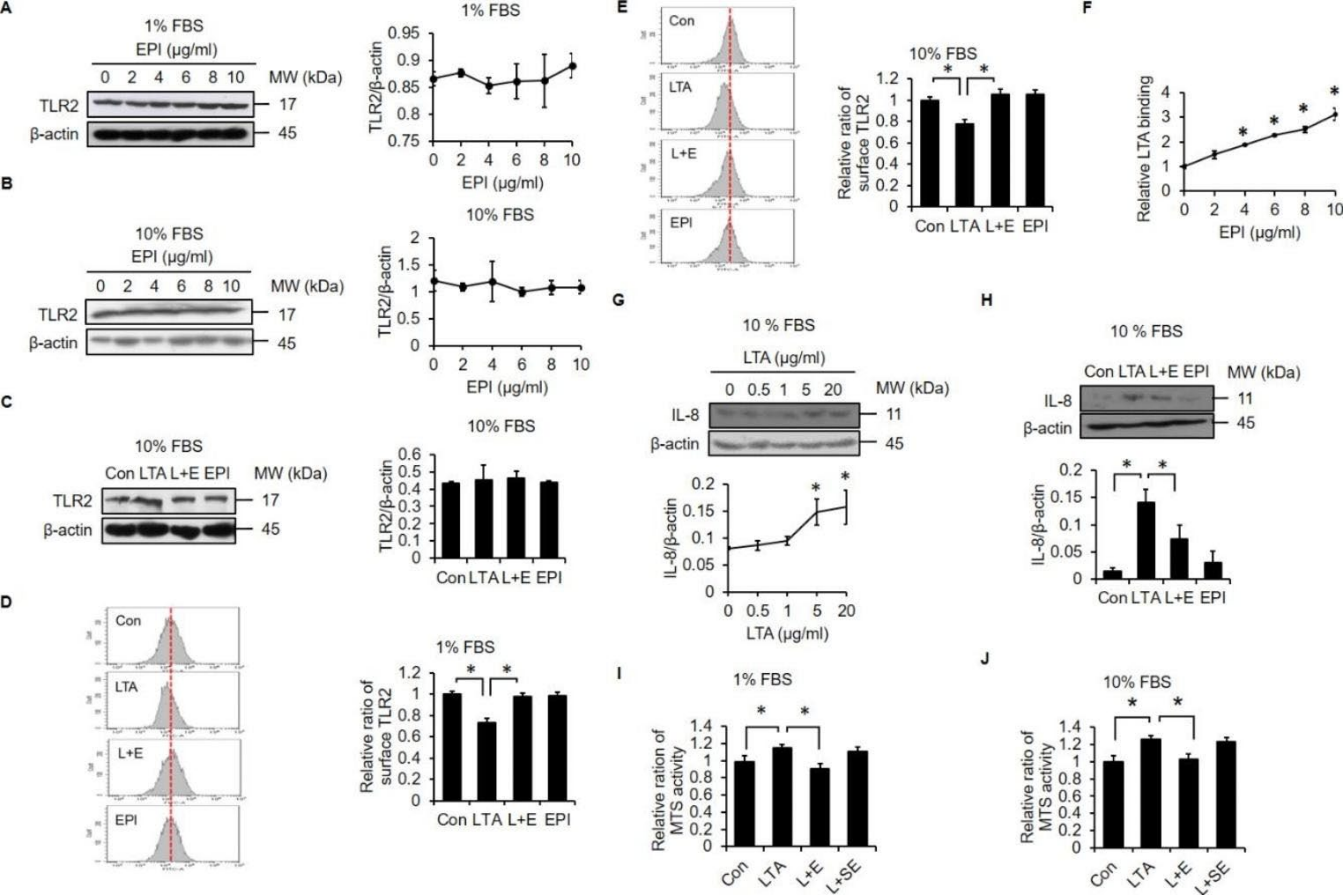
**EPI blocks LTA-induced TLR2/IL-8 signaling.** Cells were treated with increasing doses of EPI (0, 2, 4, 6, 8 and 10 µg/ml) in 1% FBS **(A)** or 10% FBS **(B)** media for 24 h. Western blot analyses and relative densitometric graphs show TLR2 protein expression levels. (C) Cells were treated with LTA (20 µg/ml) alone, EPI (6 µg/ml) alone or EPI in combination with LTA in 10% FBS medium. Expression of TLR2 was validated by western blot. Cells were treated with LTA (20 µg/ml) alone, EPI (6 µg/ml) alone or EPI in combination with LTA in 1% FBS (D) or 10% FBS (E) media. Cell surface levels of TLR2 were analyzed by flow cytometry. (F) LTA binding activity was assessed by incubating increasing doses of EPI (0, 2, 4, 6, 8 and 10 µg/ml) in LTA-coated 96-well plates. (G) Cells were treated with increasing doses of LTA (0, 0.5, 1, 5 and 20 µg/ml) in 10% FBS medium for 24 h. Expression of IL-8 was validated by western blot. (H) Cells were treated as in (E), and expression of IL-8 was analyzed by western blot. Cells were treated with LTA (20 µg/ml) alone, or LTA in combination with EPI (6 µg/ml) or SE (6 µg/ml) in 1% FBS (I) or 10% (J) FBS media. Cell viability was determined with the MTS assay. *: *P* < 0.05 compared to the control or between the indicated groups. Con: control; L + E: LTA + EPI; L + SE: LTA + SE. All experiments were independently performed at least three times

Since TLR2 is the main receptor for LTA [30], we tested whether EPI treatment might affect TLR2 expression levels. Interestingly, the total expression level of TLR2 protein was not influenced by EPI in either low (Fig. 3A) or normal serum (Fig. 3B) conditions. Furthermore, the levels of total cellular TLR2 protein were unaffected by treatments with LTA alone, EPI alone, or a combination of both (Fig. 3C). After LTA binds to TLR2, the receptor is internalized into the cytoplasm, and this internalization process is essential for stimulation of the LTA-TLR2 signaling axis [31]. Thus, we further assessed the effects of LTA and EPI on TLR2 internalization. We found that LTA stimulated TLR2 internalization in both low (Fig. 3D) and normal serum (Fig. 3E) conditions, while EPI treatment consistently blocked LTA-induced TLR2 internalization in both low and normal serum conditions. These findings suggested that EPI might interfere with TLR2 recognition of LTA, so we tested this possibility by measuring whether EPI can directly bind to LTA by ELISA. The results showed that EPI could bind to LTA-coated plates in a dose-dependent manner (Fig. 3F). It is also known that LTA stimulates NSCLC cell proliferation by upregulating IL-8 [14]. Since both EPI- and LTA-mediated activities were relatively weaker in normal serum condition, we were curious whether LTA and EPI retain their activities in normal serum conditions. Similar to the results of a previous study [14], we found that LTA treatment increased the levels of IL-8 in the normal serum condition (Fig. 3G). Importantly, we also observed that EPI treatment abolished LTA-induced elevation of IL-8 (Fig. 3H). Since the distribution of positively charged amino acid residues in EPI is crucial for its anticancer activity in other contexts [22], we wondered whether the charge distribution might impact its LTA-blocking activity. To address this question, cells were treated with LTA in the presence of EPI or scrambled EPI (SE), and the MTS assay was performed. We found that SE was unable to block LTA-induced proliferation in either low (Fig. 3I) or normal serum conditions (Fig. 3J), suggesting that the distribution of positively charged amino acid residues in EPI may be crucial for its ability to block LTA activity.

### EPI Induces Necrotic cell Death in A549 Cells

**Fig. 4:**
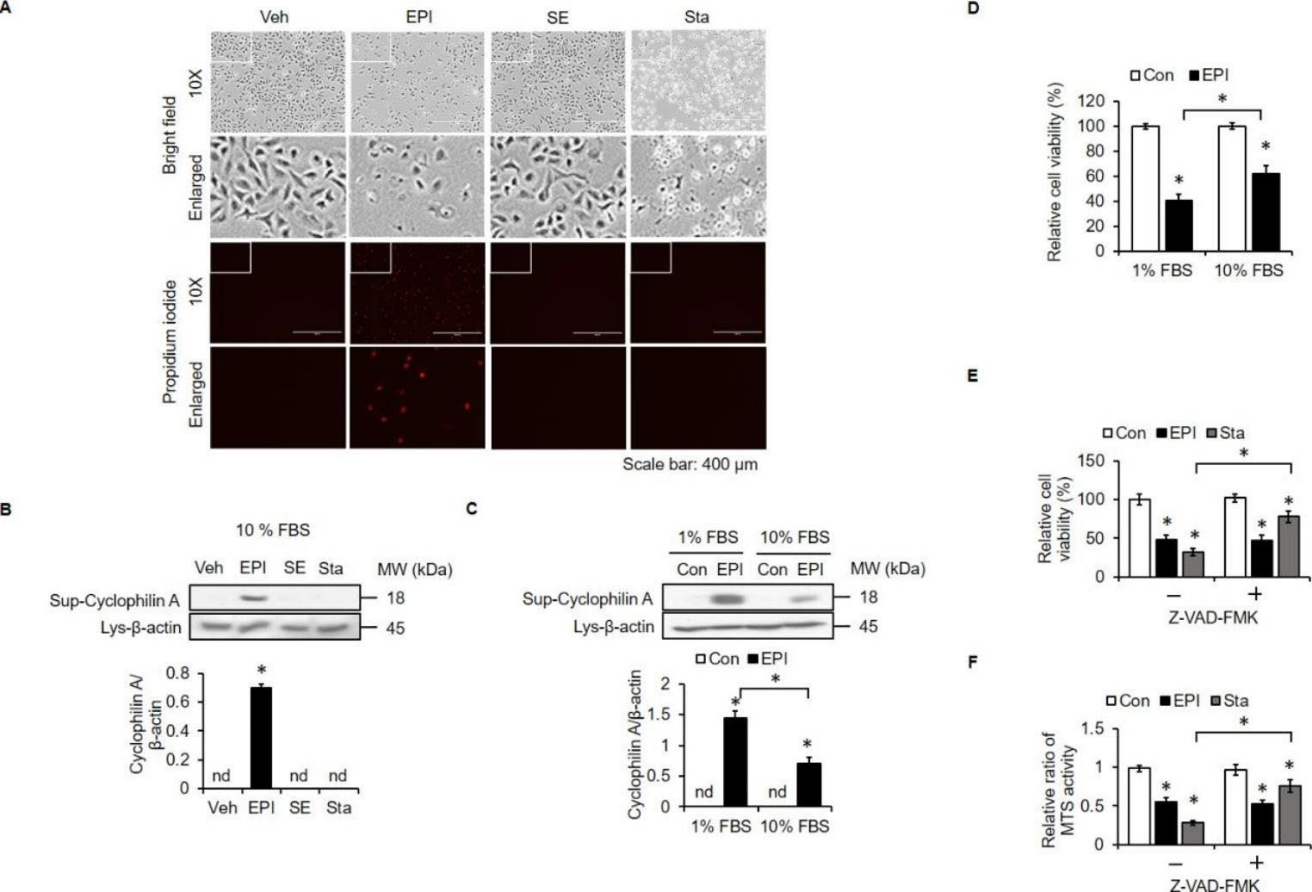
**EPI induces necrosis in A549 cells.** (**A**) Cells were treated with EPI (20 µg/ml), SE (20 µg/ml) and staurosporine (Sta; 1 µM) in 10% FBS medium for 24 h and 6 h. Cell morphology was observed under light microscopy. Scale bar: 400 μm. (**B**) Cells were treated as in (**A**). Supernatants were collected and probed with anti-cyclophilin A. Cell lysates were collected and probed with anti-β-actin. Band intensities were measured using ImageJ. (**C**) Cells were treated with EPI in 1% or 10% FBS media. Supernatants were collected and probed with anti-cyclophilin A. Cell lysates were collected and probed with anti-β-actin. Band intensities were measured with ImageJ. (**D**) Cells were treated as in (**C**). Then, cell viability was measured using trypan blue exclusion assay. Cells were preincubated with Z-VAD-FMK (100 µM) for 1 h and then treated with EPI (20 µg/ml, 24 h) or Sta (1 µM, 6 h). Cell viability was determined by trypan blue exclusion assay (**E**) and MTS assay (**F**). *: *P* < 0.05 compared to the control or between the indicated groups. Con: control; Veh: vehicle, DMSO. All experiments were independently performed at least three times

We previously found that EPI can induce necrosis in glioblastoma and synovial sarcoma cells [21, 22]. In order to evaluate whether EPI might also induce necrosis in NSCLC A549 cells, the PI uptake assay was performed on cells treated with EPI, SE, or the apoptosis inducer staurosporine in normal serum conditions. We found that intracellular PI was only observed in EPI-treated cells (Fig. 4A). Similarly, EPI treatment increased the level of necrosis marker cyclophilin A [22] in the supernatant (Fig. 4B). Next, we tested whether EPI induces necrosis in the low serum condition (Fig. 4C). The results showed that EPI-induced elevation of cyclophilin A was more pronounced in the low serum condition, suggesting that serum level can modulate EPI-mediated anticancer activity. This result was validated by an experiment showing that EPI reduces cell viability in both low serum and normal serum conditions, but the effect is much more pronounced in the low serum condition (Fig. 4D). Importantly, the pan-caspase inhibitor Z-VAD-FMK could not prevent EPI-induced decreases in cell viability (Fig. 4E) and MTS signal (Fig. 4F), suggesting that EPI is unlikely to act by stimulating apoptosis. In addition, cell viability (Fig. 4E) and MTS signal (Fig. 4F) were not affected by SE treatments.

### EPI Induces Mitochondrial Damage and ROS Production

**Fig. 5:**
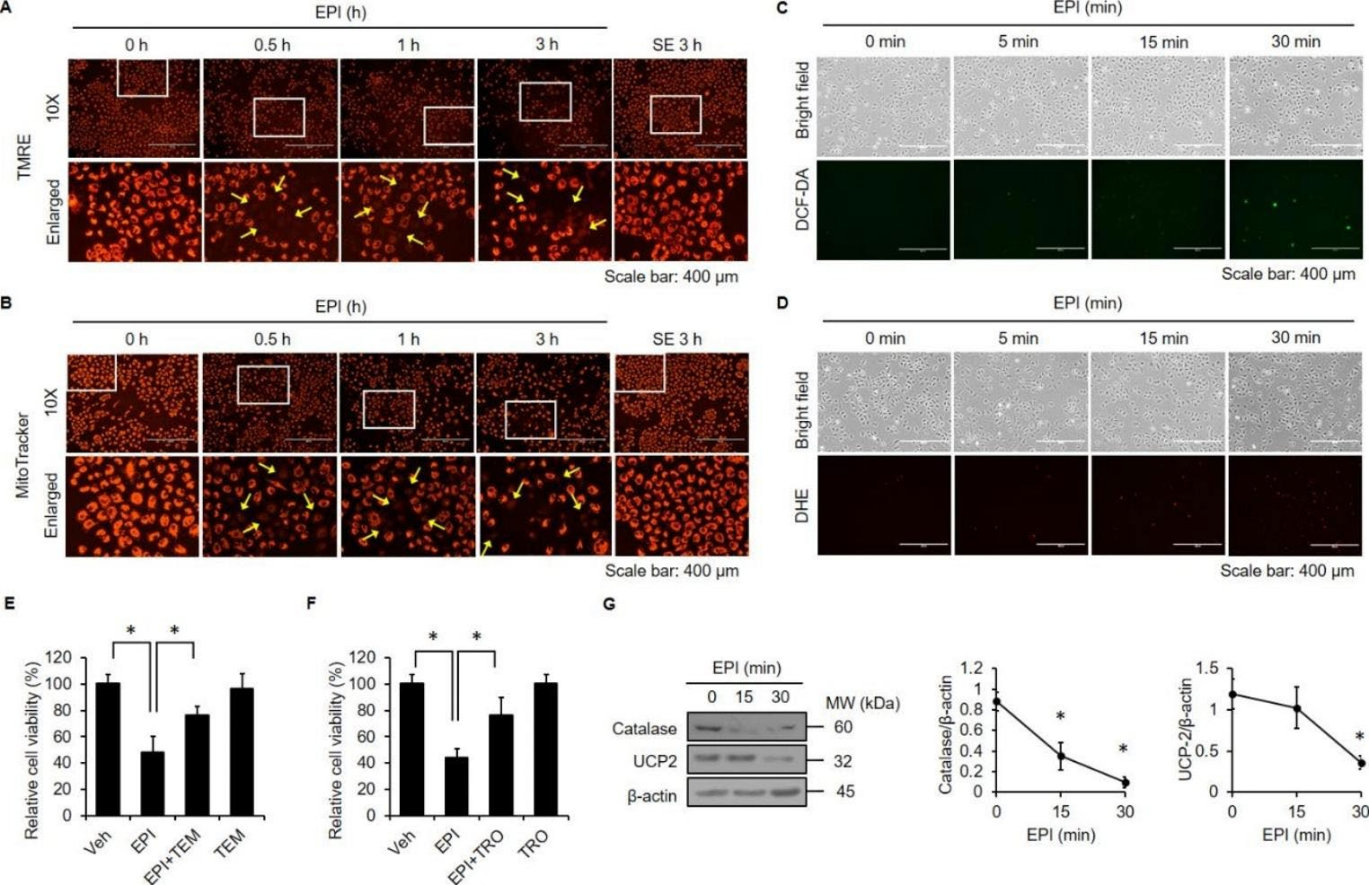
**EPI induces depolarization of mitochondria, elevation of ROS levels, and reduction of antioxidant enzyme levels.** (**A**, **B**) Cells were treated with EPI (20 µg/ml) or SE (20 µg/ml) for 3 h in 10% FBS medium. Then, cells were stained with TMRE (**A**) or MitoTracker (**B**) and observed under fluorescence microscopy. Scale bar: 400 μm. Yellow arrows indicate cells with weak TMRE or MitoTracker staining intensities. Cells were treated with EPI (20 µg/ml) for the designated times (0, 5, 15 and 30 min). Then, cells were stained with DCF-DA (**C**) or DHE (**D**). The intensities of DCF-DA and DHE were observed under fluorescence microscopy. Scale bar: 400 μm. Cells were pre-incubated with TEMPO (TEM; 150 µM) (E) or trolox (TRO; 100 µM) (**F**) for 1 h and then treated with EPI (20 µg/ml) for 24 h. Cell viability was measured using trypan blue exclusion assay. (**G**) The cells were treated with EPI (20 µg/ml) for the indicated times (0, 15 and 30 min). The western blot analysis shows the protein levels of catalase and UCP2. *: *P* < 0.05 compared to the control or between the indicated groups. Veh: vehicle, DMSO. All experiments were independently performed at least three times

We previously showed that mitochondria are major intracellular targets of antimicrobial peptides [32], so we next tested whether EPI affects the mitochondria in A549 cells. Mitochondrial status was assessed with TMRE and MitoTracker, as only functional mitochondria can be labeled by these two fluorescent mitochondrial probes [33, 34]. The TMRE (Fig. 5A) and MitoTracker (Fig. 5B) signals were lost after cells were exposed to EPI for 0.5 h, but the effect was not seen after treatment with SE. Since mitochondrial damage is correlated with reactive oxygen species (ROS) production [35], we next measured the effect of EPI treatment on ROS level. We found that the signals of fluorescent ROS indicators DCF-DA (Fig. 5C) and DHE (Fig. 5D) were upregulated after cells were exposed to EPI for 0.5 h. Moreover, the ROS scavengers TEMPO (Fig. 5E) and trolox (Fig. 5F) abolished EPI-mediated cytotoxicity, indicating a critical role of ROS in EPI-mediated cytotoxicity. Of note, we also found that EPI treatment reduced the levels of antioxidant proteins catalase and UCP2 (Fig. 5G). Together, these results demonstrate that EPI causes mitochondrial damage and increased ROS, which likely mediate its induction of necrotic cell death.

## Discussion

Chemotherapy and radiotherapy are common treatments for lung cancer. However, these treatments reduce the total leukocyte count [36, 37] and also suppress the phagocytic activity of leukocytes [38], which impairs immune response and renders patients susceptible to microbial infections. As such, *S. aureus* is the most common gram-positive bacteria identified in NSCLC patients [4, 5]. Since NSCLC patients with *Staphylococcus* sp. infections exhibit enhanced tumor metastasis [7, 39], gram-positive bacterial infections might contribute to the overall poor prognosis of NSCLC.

Although antibiotics are usually effective at preventing or controlling bacterial infections, clinical studies show that advanced NSCLC patients taking certain antibiotics (e.g., vancomycin) prior to or during chemotherapy have poor progression-free survival and overall survival [9, 40]. In addition to the direct toxicity of certain antibiotics [41], antibiotic-induced release of bacterial PAMPs might also contribute to poor clinical outcomes [11]. For instance, it was shown that LTA from *S. aureus* can enhance proliferation of NSCLC cells [14]. Thus, the use of traditional antibiotics for management of infections in NSCLC cancer patients might negatively impact clinical outcomes [42].

The levels of serum proteins and albumin significantly differ between cancer patients with cachexia and those without cachexia [43]. Furthermore, 73% of advanced lung cancer patients exhibit some level of malnutrition [44]. Malnourished patients are highly susceptible to nosocomial infection [45] and also have poor responses to cancer therapies and stronger side effects [46]. As a model of nutrient deprivation, serum deprivation in vitro can be used to partly mimic the in vivo condition [47]. Notably, LTA exhibited stronger enhancement of cell proliferation in the low serum condition (Fig. 1). Only 0.5 µg/ml LTA was sufficient to stimulate cell proliferation in the low serum condition, while a higher dose of 1 µg/ml LTA was needed to produce a similar effect in the normal serum (10% FBS) condition. These findings are similar to a previous investigation [27] that showed serum proteins could inhibit LTA-mediated activity. While it remains unclear whether the levels of TLR2 are affected by different serum concentrations, our findings suggest that *S. aureus* infection in malnourished cancer patients could lead to more prominent stimulation of cancer cell proliferation than in well-nourished cancer patients.

Similar to the results of our previous investigation [20], we found that EPI blocked LTA-mediated internalization of TLR2 (Fig. 3D and E). It is thought that the inhibitory effects of antimicrobial peptides on PAMP-induced activity are mainly mediated by direct neutralization of PAMPs rather than inhibition of receptors [48]. Then, we further demonstrated that EPI can directly interact with LTA (Fig. 3F), and the order of amino acids in EPI is crucial for this interaction (Fig. 3I and J). In addition to its LTA-neutralizing activity, EPI also kills lung cancer cells by inducing necrosis (Fig. 4). Stimulation of necrosis is a promising therapeutic strategy for various types of cancers, especially apoptosis-resistant cancers [49] like lung cancer [50]. Moreover, we found that SE had minimal to no cell killing activity (Fig. 4), suggesting that the order of amino acids residues in EPI is also crucial for its cytotoxic action. Since mitochondria are preferential intracellular targets of some antimicrobial peptides [32], we assessed the effects of EPI on mitochondria function. We found that EPI alters mitochondrial function in very short period of time, as mitochondria damage was observed after exposure to EPI for only 0.5 h (Fig. 5). This rapid action does not allow time for cancer cells to develop resistance mechanisms toward antimicrobial peptide-mediated killing. Thus, rapid action might be one of the reasons that cancer cells do not readily develop resistance to antimicrobial peptides [51]. Owing to these considerations, the use of antimicrobial peptides in cancer therapy may be preferable to the use of conventional chemotherapeutic agents.

Our previous work demonstrated that EPI can kill various pathogens known to commonly cause nosocomial infections, such as MRSA, *S agalactiae* (BCRC10787), *Streptococcus pneumonia*, and *Pseudomonas aeruginosa* (ATCC19660). The minimum inhibitory concentrations of EPI for these pathogens are respectively 6.25, 0.33, 25, and 10.7 µg/ml [52]. In this study, we show that the minimum concentration at which EPI neutralizes LTA is 4 µg/ml (Fig. 2C and D). Moreover, the minimum EPI concentrations that inhibit proliferation of lung cancer cells in low serum and normal serum are 8 µg/ml and 20 µg/ml, respectively (Fig. 2A and B). In contrast, EPI only causes hemolysis in human erythrocytes at concentrations above 50 µg/ml [26]. Thus, a therapeutic window seems to exist in which EPI should exert its multifunctional anticancer activities without causing off-target toxicity.

## Conclusion

Taken together, our findings suggest that EPI might be suitable for development as a new type of anti-lung cancer agent, owing to its cancer cell killing activity, LTA neutralizing activity, and broad-spectrum antibacterial activity. The multifaceted functions of EPI that we describe may allow the molecule or its derivatives to be used in NSCLC patients without causing many of the negative effects associated with antibiotics and chemotherapy.

## Data Availability

The raw data supporting the conclusions of this article will be made available by the authors, without undue reservation.
